# Identification of a TPX2-Like Microtubule-Associated Protein in *Drosophila*

**DOI:** 10.1371/journal.pone.0028120

**Published:** 2011-11-30

**Authors:** Gohta Goshima

**Affiliations:** Division of Biological Science, Graduate School of Science, Nagoya University, Furo-cho, Chikusa-ku, Nagoya, Japan; University of Texas-Houston Medical School, United States of America

## Abstract

Chromosome segregation during mitosis and meiosis relies on the spindle and the functions of numerous microtubule-associated proteins (MAPs). One of the best-studied spindle MAPs is the highly conserved TPX2, which has been reported to have characteristic intracellular dynamics and molecular activities, such as nuclear localisation in interphase, poleward movement in the metaphase spindle, microtubule nucleation, microtubule stabilisation, microtubule bundling, Aurora A kinase activation, kinesin-5 binding, and kinesin-12 recruitment. This protein has been shown to be essential for spindle formation in every cell type analysed so far. However, as yet, TPX2 homologues have not been found in the *Drosophila* genome. In this study, I found that the *Drosophila* protein Ssp1/Mei-38 has significant homology to TPX2. Sequence conservation was limited to the putative spindle microtubule-associated region of TPX2, and intriguingly, D-TPX2 (Ssp1/Mei-38) lacks Aurora A- and kinesin-5-binding domains, which are highly conserved in other animal and plant species, including many insects such as ants and bees. D-TPX2 uniformly localised to kinetochore microtubule-enriched regions of the metaphase spindle in the S2 cell line, and it had microtubule binding and bundling activities *in vitro*. In comparison with other systems, the contribution of D-TPX2 to cell division seems to be minor; live cell imaging of microtubules and chromosomes after RNAi knockdown identified significant delay in chromosome congression in only 18% of the cells. Thus, while this conserved spindle protein is present in *Drosophila*, other mechanisms may largely compensate for its spindle assembly and chromosome segregation functions.

## Introduction

The spindle is a microtubule-based structure essential for segregating chromosomes in eukaryotes [1], [2]. In most animal cell types, centrosomes play a dominant role in spindle microtubule nucleation. However, non-centrosomal pathways, such as augmin-dependent microtubule amplification and chromosome-induced microtubule nucleation, are also present in the cells that possess centrosomes [1], [3], [4], [5]. A main player in the latter pathway is a GTPase Ran, whose activator, RCC1, is concentrated on the chromosomes. Thereby, Ran locally activates several downstream proteins required for spindle assembly, such as the microtubule-stabilising protein HURP and the crosslinker kinesin-14 [1], [6], [7], [8], [9].

TPX2 is one of the best-characterised targets of Ran. This conserved microtubule-associated protein (MAP) was originally identified as a protein required for targeting kinesin-12 (Xklp2) to the spindle pole in *Xenopus* egg extracts [10]. Subsequent functional studies have established that TPX2 is essential for spindle assembly, in particular for spindle pole organisation in a variety of cell types [11], [12], [13], [14], [15], [16], [17], [18]. TPX2 is imported into the nucleus by importin binding during interphase and is subsequently activated by the removal of importin by RanGTP following nuclear envelope breakdown (NEBD) [13], [19], [20]. Several conserved domains have been identified in TPX2, including regions responsible for nuclear localisation (i.e. importin binding), activation of the Aurora A kinase, microtubule nucleation and stabilisation, and kinesin-5 binding. The mechanism of Aurora A activation has been elucidated at the atomic level [21], and structure-function studies have clarified the importance of this domain in spindle size control [14] and suggested its role in spindle assembly itself [22] (the latter has been disputed [20]). Although the microtubule nucleation activity has been shown *in vitro*, the physiological significance of this activity has been controversial [20], [23], [24]; lack of microtubule generation in the absence of TPX2 in cells has been suggested to be due to stabilisation defects rather than nucleation problems [23]. TPX2 is most concentrated at spindle poles partly due to motor-dependent transport, but it is also localised all along the spindle microtubules [10], [14], [20], [24]. Although the organisation of these domains is generally conserved among multicellular organisms, there are 2 exceptions. In *Caenorhabditis elegans*, the TPX2-like protein (TPXL-1) appears to be missing all the conserved domains other than that responsible for Aurora A activation [12]. Another, perhaps more mysterious issue is that homologous proteins to TPX2 have not been found in the genome of *Drosophila melanogaster*, one of the most popularly used model animal species for cell division research, although HURP and kinesin-14, 2 other Ran targets have been identified as the nuclear (interphase) and spindle (mitosis) proteins [25], [26].

The *Drosophila* Ssp1/Mei-38 gene was identified in 2 independent studies. In a genome-wide RNAi screen for spindle morphology, knockdown of this gene elevated the percentage of spindles with slightly abnormal morphology, such as shorter, monastral bipolar or monopolar spindles [27]. On the other hand, genetic screening by Baker and Carpenter (1972) identified an allele of *mei-38* for elevated levels of X chromosome nondisjunction in female flies [28], and recent cloning by Wu et al. (2008) revealed that Mei-38 is identical to Ssp1 [29]. The null mutant exhibits defects in meiotic spindle morphology in female flies. However, although slight spindle organization defects are seen in mitotic cells in the larval brain, the mutant is completely viable with no noticeable defects. Both studies localised this protein to spindle microtubules, consistent with its role in spindle assembly, but found homologous proteins only in Diptera (the order that encompasses flies and mosquitoes) [27], [29]. Whether this protein binds to microtubules directly remains unknown.

This study began with further characterisation of Ssp1/Mei-38 in the *Drosophila* S2 cell line, hypothesising that this unique protein in Diptera may provide an insight into the unique mechanism of spindle assembly in these insect species. Unexpectedly, it was found that Ssp1/Mei-38 has significant sequence similarity to TPX2, but that the domain organisation is very unique among TPX2 family proteins in other species. Function and evolution of this unique TPX2-like protein in *Drosophila* are discussed.

## Results

### Ssp1/Mei-38 is homologous to the putative microtubule-binding domain of TPX2

**Figure 1:**
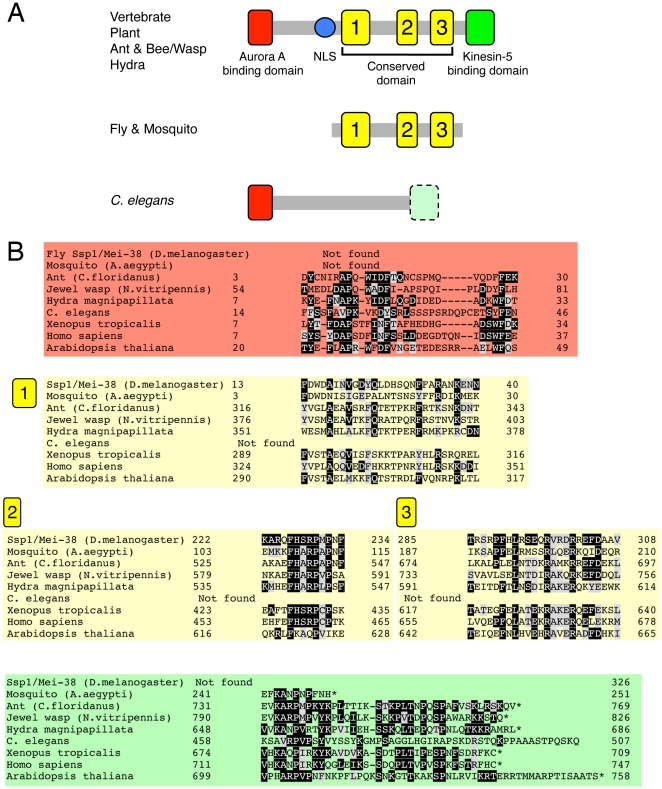
Ssp1/Mei-38, a putative TPX2 homologue in *Drosophila*. Domain organisation (A) and sequence alignment (B) of TPX2 family proteins. The conserved regions are coloured (red, green, and yellow). The putative kinesin-5-binding domain in *C. elegans* (light green) was only detected by manual inspection of the sequences, which might not be functionally significant.

In the course of this project, a PSI-BLAST was applied to the full-length Ssp1/Mei-38 protein. Intriguingly, vertebrate TPX2, a well-known MAP was listed as a protein with sequence similarity. Further comparisons of Ssp1/Mei-38 and other TPX2 sequences by using the MEME search as well as manual inspection revealed 3 conserved domains between Ssp1/Mei-38 and TPX-2 (Figure 1), among which the second domain has the highest conservation. No proteins more similar to TPX2 were found in the *Drosophila* genome. Sequence conservation of D-TPX2 (Ssp1/Mei-38) was restricted to the middle portion of vertebrate TPX2, which is a part of the putative microtubule-localising site [20]. D-TPX2 lacks the domains responsible for Aurora A-binding (N-terminus), nuclear localisation (∼250 a.a.), and kinesin-5/Eg5-binding (C-terminus), which have been under active investigation. Surprisingly, lack of Aurora-A and kinesin-5 -binding domains is not a generally conserved feature among insect species; those domains are found in TPX2 of jewel wasps, honey bees, and ants, which suggests that Diptera, including flies and mosquitoes, lost the domain fairly recently during evolution (Figure 1).

### D-TPX2 may only partially contribute to chromosome segregation in S2 cells

**Figure 2:**
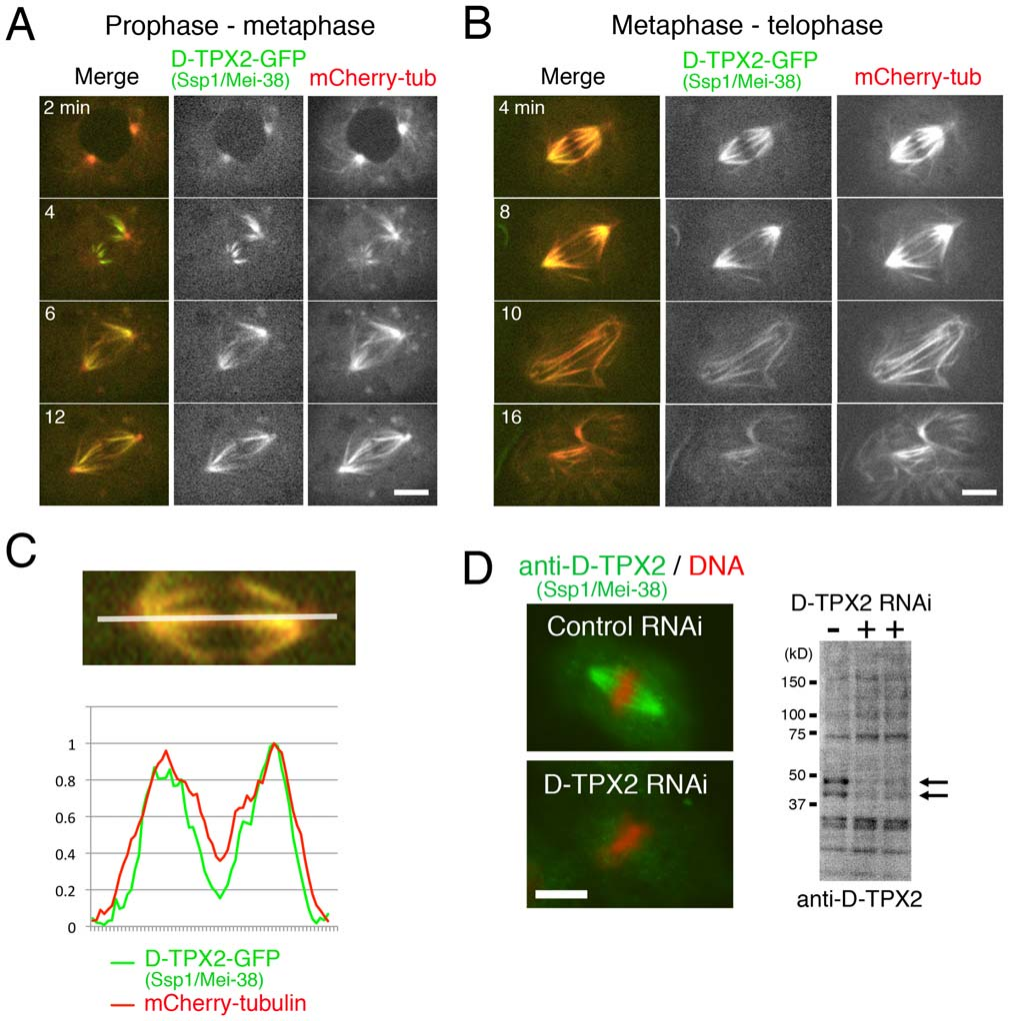
Enrichment of D-TPX2 on kinetochore microtubules in early mitosis. (**A**) Localisation of D-TPX2-GFP (green) from prophase to metaphase. (**B**) Localisation of D-TPX2-GFP (green) from metaphase to telophase. (**C**) Enrichment of D-TPX2-GFP at the kinetochore microtubules compared to interpolar microtubules that are interdigitated at the middle. (**D**) (left) Localisation of endogenous D-TPX2 by immunostaining using a polyclonal antibody. The specificity of the antibody was confirmed by immunostaining of RNAi-treated cells. (right) Immunoblotting using anti-D-TPX2. Two D-TPX2 RNAi samples were derived from the different dsRNA sequences used in Goshima et al. (2007). The antibody recognised 2 bands (arrows) that disappeared after RNAi; one of them might be a degradation product or reflect post-translational modification of D-TPX2. Bars, 5 µm.

Previous loss-of-function studies on D-TPX2 were based upon fixed cells. In this study, the mitotic phenotype was assessed by time-lapse imaging of GFP-tubulin and Histone-mRFP after RNAi knockdown of D-TPX2 in S2 cells (Video S1). The noticeable defects reproducibly seen in D-TPX2 RNAi cells were subtle; 82% of the cells progressed through mitosis without any noticeable defects in 28±10 min (n = 80), which is not significantly different from control cells (30±16 min, n = 77). The remaining 18% of the cells took an extremely long time to undergo mitosis (>60 min) with misaligned chromosomes (99±49 min, n = 14). This scale of delay was rarely seen in controls cells (2%) and was reproduced in 4 independent experiments. The mild phenotype might be due to 15% of D-TPX2 proteins remaining after 5-day RNAi (see Figure 2D). However, even when RNAi treatment was extended to 10 days, only the mild phenotype was observed (9 out of 60 cells had >60 min mitosis; mitotic duration of the remaining 51 cells was 24±9 min). Therefore, a more probable interpretation would be that D-TPX2 plays only a minor role in chromosome segregation in S2 cells, as does so in other somatic cells in the fly [29].

It was assumed that the phenotype might become more evident in a sensitised condition; however, double RNAi experiments have not revealed any additive effects thus far. For example, no synthetic phenotypes have been observed after double or triple RNAi with Cnn, required for centrosome formation, the Subito kinesin, for which genetic interactions have been described [29], or the other 2 Ssp1/Mei-38-like proteins (CG15395 and CG5781) in *Drosophila*, which are expressed exclusively in the testis according to the available database (FlyAtlas; [30]).

Taken altogether, it is strongly suggested that D-TPX2 plays a minor role in mitotic spindle formation and chromosome segregation, which differs from TPX2 in other species, for which dramatic mitotic phenotypes have been observed upon depletion.

### D-TPX2 is a spindle microtubule-associated protein

D-TPX2 has been shown to localise to spindle microtubules by using single-time point image acquisition of D-TPX2-GFP in S2 cells and by immunostaining of D-TPX2-HA in meiotic cells [27], [29]. In the current study, D-TPX2-GFP localisation was monitored during mitosis in living S2 cells that co-express mCherry-tubulin. Furthermore, endogenous D-TPX2 localisation was determined by immunostaining (Figure 2 and Video S2).

D-TPX2-GFP was not found in the nucleus but colocalised with microtubule organising centres (MTOCs) and emanated microtubules during prophase (Figure 2A, 2 min). Upon NEBD, the signals at the MTOC became less evident, and instead, spindle microtubules, perhaps including kinetochore microtubules, were heavily decorated by D-TPX2-GFP during prometaphase and metaphase (4–12 min). In metaphase, the D-TPX2-GFP signal was more clearly observed on the half-spindle regions that are abundant in kinetochore microtubules in S2 cells than at the central regions of the spindle, which are presumably dominated by interpolar microtubules that interdigitate each other in an antiparallel orientation (Figure 2B, 4 min, and 2C). These localisations are slightly different to vertebrates where TPX2 accumulates to a greater extent near the spindle pole. However, spindle localisation and exclusion from the central region was confirmed using a polyclonal antibody that recognises endogenous D-TPX2 in S2 cells (Figure 2D), and similar observations have been made by D-TPX2-HA immunostaining in female meiotic spindles [29]. Thus, D-TPX2 is a spindle-associated protein that likely binds to kinetochore microtubules more preferentially than to antiparallel interpolar microtubules during pre-anaphase. During anaphase, D-TPX2 was uniformly localised on the spindle microtubules (Figure 2B).

### A conserved domain is required for microtubule association

**Figure 3:**
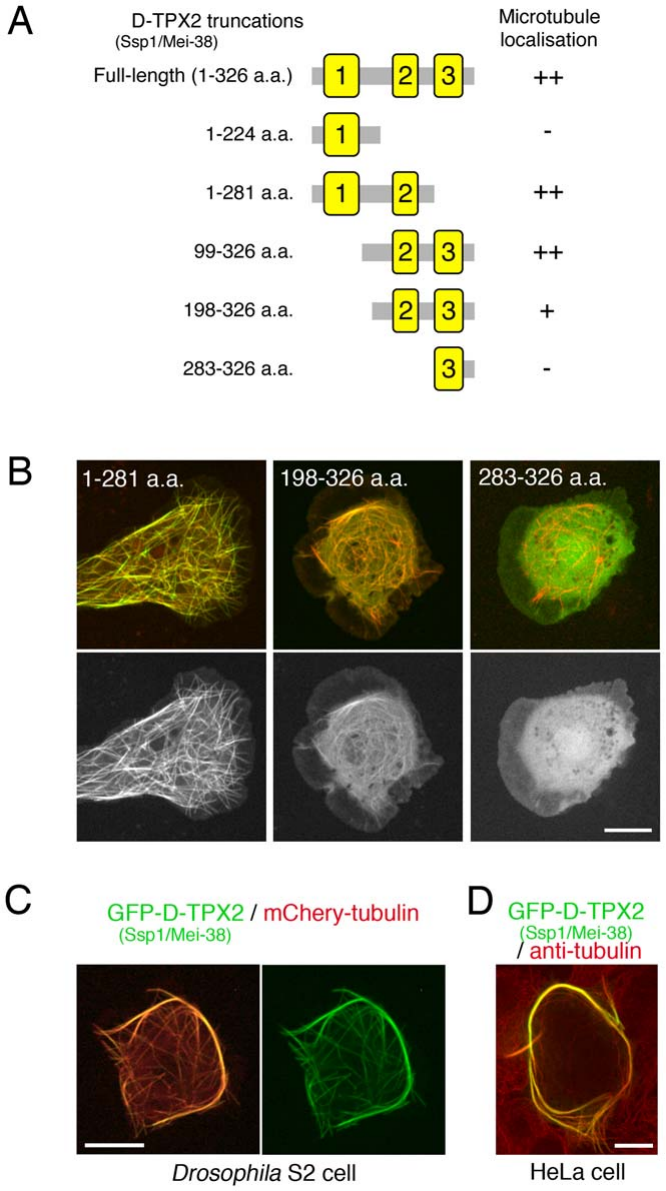
Conserved domain 2 of D-TPX2 is critical for microtubule localisation. (**A**) Truncated constructs of D-TPX2. The 3 conserved domains are coloured yellow. (**B**) Localisation of truncated D-TPX2 proteins tagged with GFP in S2 cells (green). mCherry-tubulin (red) was co-expressed. (**C, D**) Overexpression of full-length D-TPX2-GFP in the S2 or HeLa cytoplasm induced microtubule bundling. Bars, 10 µm.

In order to determine the domain(s) responsible for microtubule association, a series of truncated D-TPX2 proteins tagged with GFP were expressed in S2 cells (Figure 3A). It was found that the most conserved domain (domain 2) is required for microtubule localisation (Figure 3B). In this ectopic expression experiment, it was also revealed that overexpression of D-TPX2 in interphase causes bundling of cytoplasmic microtubules (Figure 3C). Similar bundling effects were also seen when D-TPX2 was overexpressed in human HeLa cells (Figure 3D).

### D-TPX2 binds to and bundles microtubules *in vitro*

**Figure 4:**
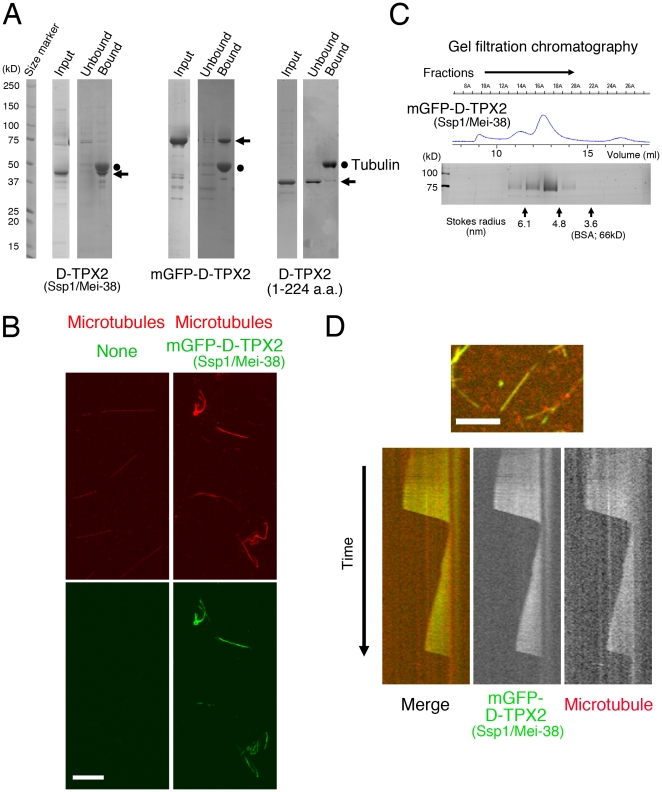
D-TPX2 is a microtubule-associated protein. (**A**) Microtubule cosedimentation assay with purified D-TPX2 (full-length, 2 µM), mGFP-D-TPX2 (full-length, 2 µM), and a D-TPX2 fragment (1–224 a.a., 5 µM) that lacks conserved domains 2 and 3 (see Figure 1). These proteins were expressed and purified using the baculovirus expression system (Input). Proteins were mixed with 2.5 µM taxol-stabilised microtubules, followed by centrifugation. Arrows indicate D-TPX2 proteins. (**B**) Microtubule bundling assay with purified mGFP-D-TPX2 (full-length, 0.2 µM) and taxol-stabilised microtubules (4 µM). No protein was added to the control sample (left). Bar, 10 µm. (**C**) Gel filtration chromatography followed by SDS-PAGE and silver staining of purified mGFP-D-TPX2. UV absorbance was also monitored (blue line). Ferritin (440 kD; Stokes radius, 6.1 nm), aldolase (158 kD; 4.8 nm), and BSA (67 kD; 3.6 nm) were used as size markers. mGFP-D-TPX2 was fractionated before BSA, the molecular weights of both being around 70 kD. (**D**) D-TPX2 binds uniformly to growing or shrinking microtubules *in vitro*. Rhodamine-labelled tubulin (9 µM) was mixed with 400 nM mGFP-D-TPX2 (full-length) in this experiment. Bar, 5 µm.

Strong accumulation of D-TPX2 on the spindle microtubules and bundling of cytoplasmic microtubules upon D-TPX2 overexpression suggested that D-TPX2 directly binds to and bundles microtubules. To test this hypothesis, full-length D-TPX2, monomeric GFP (mGFP)-tagged D-TPX2, and truncated D-TPX2 (1–224 a.a.) that has no spindle localisation were expressed and purified using the baculovirus expression system. The microtubule co-pelleting assay and bundling assay showed that D-TPX2 indeed binds to and bundles taxol-stabilised microtubules *in vitro* (Figure 4A and 4B). Gel filtration chromatography showed that mGFP-D-TPX2 has a Stokes radius of 5.4 nm, which is significantly larger than that of a monomeric protein of similar size (bovine serum albumin), suggesting that D-TPX2 might form a multimer (Figure 4C). Finally, the *in vitro* microtubule polymerisation assay that specifically monitors single dynamic microtubules revealed that mGFP-D-TPX2 localises to the growing and also shrinking microtubules (Figure 4D and Video S3). These results indicated that D-TPX2 is a microtubule binding protein that also has a bundling activity, a conserved feature among TPX2 family proteins [31].

### The extra C-terminal region is required for TPX2 function in human cells

**Figure 5:**
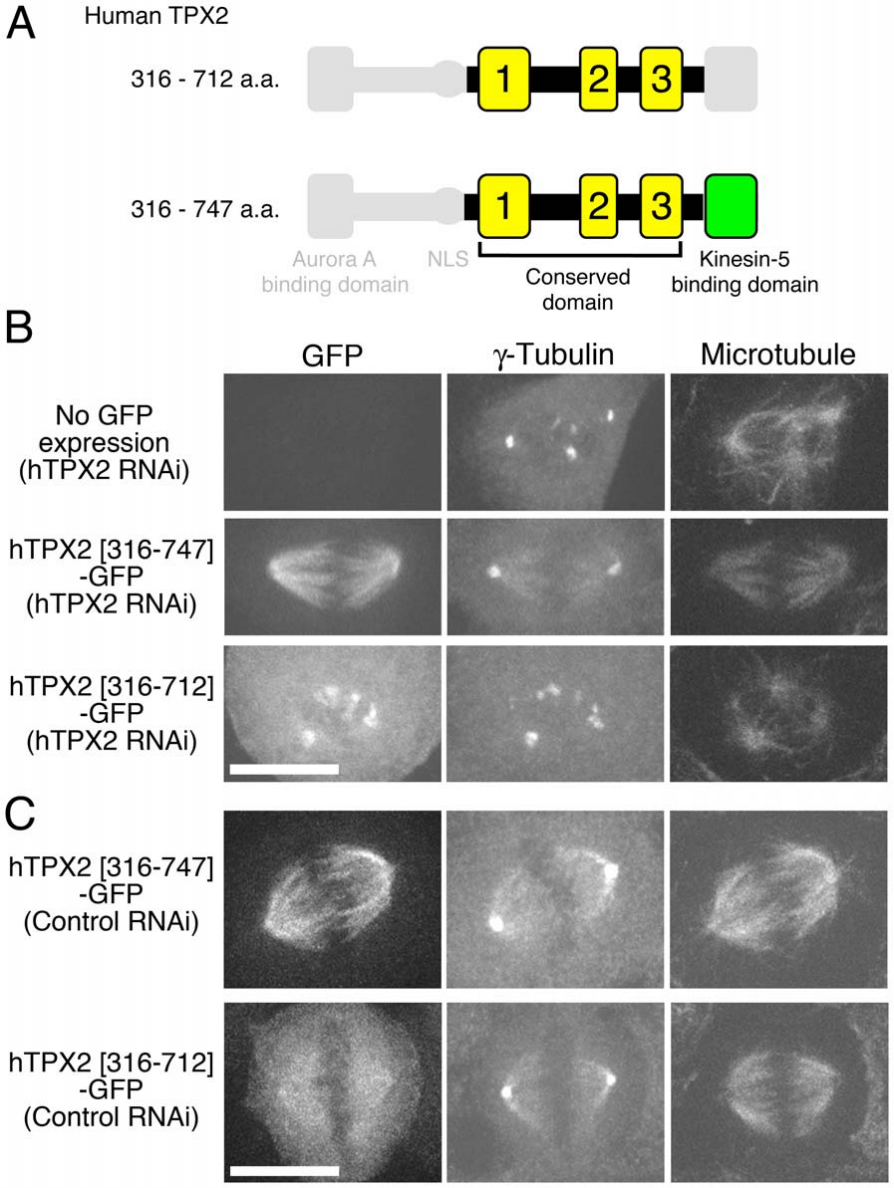
Human TPX2 requires the C-terminal domain, lacking in *Drosophila* D-TPX2, for spindle function. (**A**) The truncated human TPX2 (hTPX2) used in this experiment. (**B**) Mitotic spindles observed after RNAi knockdown of endogenous TPX2 in the presence or absence of truncated hTPX2-GFP expression. Bipolarity of the spindle was largely restored by expressing hTPX2-GFP (316–747 a.a.) but not hTPX2-GFP (316–712 a.a.). (**C**) Spindle localisation of hTPX2-GFP (316–747 a.a.) and hTPX2-GFP (316–712 a.a.) in the control cells. Cytoplasmic signals were also detected for the shorter fragment. Z-stack images (1-µm interval, 13 sections) were acquired by spinning-disk confocal microscopy, and the projected images are displayed in (B) and (C). Bars, 10 µm.

Studies on truncated vertebrate TPX2 have been performed, but the role of the minimum conserved region identified in this study has not been investigated [14], [20]. To gain insight into the function of the conserved domain in human TPX2, the corresponding fragment of human TPX2 (316–712 a.a.) was cloned into an expression vector (Figure 5A). As a control, the fragment that further contains the domain binding to kinesin-5 [18] and possibly also kinesin-12 [16] was used (316–747 a.a.). Consistent with previous rescue experiments performed in U2OS cells or *Xenopus* egg extracts, in which a fragment lacking the Aurora A binding site was expressed in the absence of endogenous TPX2 [14], [20], the expression of the longer fragment (316–747 a.a.) partially rescued the spindle defect in HeLa cells; TPX2 RNAi led to multipolar spindle formation with apparently sparse microtubules around chromosomes (only 20% was bipolar; n = 49) but expression of hTPX2 (316–747 a.a.) suppressed this phenotype (69% bipolar; n = 16) (Figure 5B). In contrast, the multipolar phenotype was not rescued by expressing the shorter fragment hTPX2 (316–712 a.a.) (13% bipolar; n = 15). The shorter fragment localised to the spindle microtubules more weakly than the longer one (Figure 5C). Thus, the critical domain of hTPX2 is suggested to reside outside the conserved domain. The C-terminus of hTPX2 may function through kinesin-5 interaction as suggested recently for murine TPX2 [32], but may also do so via an increase in microtubule-binding affinity.

## Discussion

### TPX2-like proteins in *Drosophila*

This study originated with the basic characterisation of a possible fly-specific spindle protein, anticipating that a unique mechanism of *Drosophila* spindle formation might be uncovered. However, during the course of this project, the protein was found to have weak but significant homology to TPX2, one of the best-characterised spindle MAPs. Although the loss-of-function phenotype of D-TPX2 (Ssp1/Mei-38) is much more subtle than expected from TPX2 in other species, and important domains for TPX2 are missing in D-TPX2, I believe that D-TPX2 (Ssp1/Mei-38) is the fly orthologue of TPX2 for a few reasons. First and most importantly, when full-length D-TPX2 is used as the query of the PSI-BLAST search, TPX2 proteins from several species are listed as hits. Conversely, when human TPX2 is used as the query, D-TPX2 family proteins appear as the top hits among Drosophilidae species (i.e. more similar proteins to TPX2 have not been found in Drosophilidae). Second, D-TPX2 localises to spindle microtubules like other TPX2 proteins and, albeit weakly, functions in spindle morphogenesis in somatic as well as meiotic cells. Finally, the conserved region between D-TPX2 and human TPX2 has the microtubule-binding and bundling activities. In particular, it was revealed that the most conserved domain, domain 2, in D-TPX2 is critical for microtubule binding.

The sequence comparison of TPX2 family proteins may provide an evolutionary insight into this conserved protein. The ‘full-form’ of TPX2 is present in various animal and plant species but not in yeasts. However, certain animal lineages have lost or dramatically altered some domains; *C. elegans* might have considerably altered the amino acid sequences of all domains other than the Aurora A activation domain [12] and Diptera has lost all but the microtubule-binding domain. It is possible that these missing domains in Diptera were attached to other genes in the genome, although my BLAST searches have not identified such a case. Interestingly, D-TPX2-like genes cannot be found in the genome of *Anopheles gambiae*, the malaria mosquito belonging to Diptera. This is an unusual case, since another mosquito species *Aedes aegypti* does have a D-TPX2-like protein (i.e. ‘shorter’ form). This might be because D-TPX2 is encoded at the unread genome region of *A. gambiae*; however, another viable idea, based also upon the fact that *Drosophila* can survive and produce progeny without D-TPX2, is that *A. gambiae* completely lost this gene family from the genome recently. In any case, it is suggested that Diptera possesses, if any, a highly different form of TPX2 to other species.

### D-TPX2 function in cell division

A previous study using a null mutant showed that D-TPX2 plays a role in spindle formation and chromosome alignment during female meiosis I, where >50% of the spindles showed certain types of abnormality, such as ‘pole not tapered’ (21%), ‘tubulin weak between poles’ (21%), or ‘monopolar’ (17%) [29]. Retrospectively, these phenotypes are generally consistent with the defects observed after TPX2 depletion in vertebrate systems. However, 43% of the spindle appears normal in the absence of D-TPX2, and the mutant is fertile [28], [29]. Moreover, the phenotype is even milder in somatic cells, such as S2 cells ([27] and this study) and cells in larval brains; and the null mutant has no noticeable effect on viability [29]. These results strongly suggest that TPX2 in *Drosophila* makes minor contributions to cell division compared to other systems.

It is therefore reasoned that *Drosophila* has developed alternative mechanisms that compensate TPX2 function. For example, centrosomal microtubule nucleation may be robust enough to produce enough microtubules in *Drosophila* cells. Alternatively, *Drosophila* may have sufficient amounts of microtubule stabilisers other than TPX2 during mitosis. A similar scenario might hold true in certain cell types in vertebrates; there is a report using *Xenopus* egg extracts, in which TPX2 depletion phenotype is largely compensated by excess addition of XMAP215, a microtubule polymerase [23].

What exactly is ‘the minor role’ D-TPX2 plays during spindle assembly? The localisation and biochemical data suggest that D-TPX2 might be primarily involved in the action toward kinetochore microtubules, possibly helping their stable bundling. It has been suggested that kinetochore microtubule defects are not only associated with chromosome misalignment but also with spindle morphology defects (e.g. [33], [34]).

In summary, this study identified a uniquely-evolved TPX2-like protein in *Drosophila*. Loss-of-function phenotypes in S2 cells as well as other cell types in *Drosophila* strongly suggest that D-TPX2 only contributes a minor amount to spindle integrity and chromosome segregation compared to TPX2 in other cell types reported so far; thus, even a ‘must-be-essential’ protein could be dispensable for spindle assembly in certain cell types/organisms. This study therefore reinforces the idea that spindle assembly is driven by multiple mechanisms and the extent of usage of each mechanism varies in different cell types. It is further suggested that the presence of redundant mechanisms allows dramatic changes to the structure of a mitotic gene itself during evolution.

## Materials and Methods

### Cell culture, transfection, and RNAi

*Drosophila* S2 cells (obtained from the UCSF cell culture facility [35]) were cultured and RNAi was performed as previously described [36], [37]. Plasmid transfection was done with Cellfectin II (Invitrogen). RNAi-treated cells were observed at day 4, 5, or 10. For the day 10 observation, cells that were treated with dsRNA at day 0, 3, and 6 were observed. HeLa cells [38] were cultured in DMEM supplemented with 10% serum. Transfection was performed using the Jet-PEI reagent (Polyplus transfection) and RNAi was carried out using the RNAi max (Invitrogen). siRNA for TPX2 is described in [14], and RNAi-treated cells were observed after 30 h of treatment.

### Microscopy

At the end of the RNAi treatment, the S2 cells were resuspended and transferred to glass-bottomed, concanavalin A (Con-A)-coated plates with multiple wells. Images were acquired every 2 min for several hours at 25°C by using a wide-field microscope TE2000 (Nikon) attached with 40× 1.30 NA oil immersion lens and a CCD camera (Micromax; Roper). In order to keep the cells in focus during the long-term imaging, the perfect focus system (PFS; Nikon) was activated throughout the imaging, and control cells and RNAi-treated cells were plated side-by-side. GFP localisation was determined using a spinning-disk confocal microscope (CSU-X1; Yokogawa) with 100× 1.45 NA lens and an EMCCD camera (ImagEM; Hamamatsu). For HeLa cells, immunostaining was performed with anti-tubulin (YOL1/34) and anti-γ-tubulin (GTU-88) staining after paraformaldehyde fixation, followed by imaging with a wide-field or spinning-disk microscope described above.

### Protein purification

His-D-TPX2, His-D-TPX2 (1–224 a.a.) and His-mGFP-D-TPX2 were expressed in Sf21 cells that were infected with baculovirus. Infected cells were treated with the extraction buffer (1× MRB80 (80 mM K-PIPES pH 6.8, 4 mM MgCl_2_, and 1 mM EGTA), 100 mM KCl, 1% Triton, protease inhibitors), followed by affinity purification with Ni-NTA beads. After washing 3 times with the washing buffer (1× MRB80, 500 mM KCl), proteins were eluted with 1× MRB80 with 200 mM imidazole and 100 mM KCl, followed by dialysis at 4°C by using the stock buffer (1× MRB80, 100 mM KCl, 20% Glycerol). Proteins were then flash frozen into liquid nitrogen and kept at −80°C. To obtain the rabbit polyclonal antibody against D-TPX2, His-D-TPX2 (full-length) was expressed in the *E. coli* BL21-AI strain and purified by Ni-NTA in a denatured condition. Gel filtration chromatography was performed using the BioLogic DuoFlow system (Biorad) with a Superdex 200 10/300 GL column (GE healthcare).

### Microtubule binding and bundling assays

For the microtubule cosedimentation assay, tubulins extracted from pig brains were polymerised in the presence of 2 mM GTP, 1 mM DTT, and 20 µM taxol at 37°C. Microtubules and recombinant D-TPX2 proteins were mixed at room temperature for 15 min in 1× MRB80 with 50 mM KCl and 10% glycerol, followed by centrifugation (106,000 *g*, 15 min at 25°C). Supernatants (unbound) and pellets (bound) were boiled with the SDS-containing buffer and analysed by SDS-PAGE and Coomassie blue staining. The microtubule bundling assay was performed with taxol-stabilised microtubules that were partially labelled with Rhodamine. Microtubules (4 µM) and recombinant mGFP-D-TPX2 (0.2 µM) were mixed at room temperature for 15 min in 1× MRB80 with 50 mM KCl, followed by fixation with 1% glutaraldehyde. Images were acquired by using a spinning-disk confocal microscope. The *in vitro* microtubule polymerisation assay was performed as previously described [39], [40]. In brief, microtubule seeds were made by tubulin polymerisation in the presence of GMP-CPP and attached to the glass surface. Unlabelled tubulins, Rhodamine-labelled tubulins, and mGFP-D-TPX2 were applied to the glass. Experiments were performed at 25°C, and imaging was performed with spinning-disk confocal microscopy.

## Supporting Information

Video S1 **Chromosome misalignment and mitotic delay after D-TPX2 RNAi in S2 cells.** GFP-tubulin (green) and histone H2B-mCherry (red) images were acquired every 2 min by wide-field microscopy with 40× 1.30 NA objective lens. Two cells treated with D-TPX2 RNAi had >60 min mitosis. Bar, 5 µm.
(MOV)

Video S2 **Spindle localisation of D-TPX2 during mitosis.** D-TPX2-GFP (green) and mCherry-tubulin (red) were imaged every 30 or 60 s by spinning-disk confocal microscopy. Enrichment of D-TPX2-GFP on kinetochore microtubule bundles is evident from prometaphase to metaphase. Bar, 5 µm.
(MOV)

Video S3 **D-TPX2 binds to dynamic microtubules** ***in vitro*** **.** Purified mGFP-D-TPX2 (green) was localised on dynamic microtubules (red) in the *in vitro* microtubule polymerisation assay.
(MOV)
